# The Lost Tribe: The Importance of Health Care Staff and Service Design During COVID-19

**DOI:** 10.1017/dmp.2021.77

**Published:** 2021-03-25

**Authors:** Chlöe N. Schooling, Norbert Gyenge, Visakan Kadirkamanathan, James J. P. Alix

**Affiliations:** 1 Sheffield Institute for Translational Neuroscience, University of Sheffield, UK; 2 Department of Automatic Control and Systems Engineering, University of Sheffield, UK; 3 Information Technology Services, University of Sheffield, UK

**Keywords:** COVID-19, Disaster Planning, Delivery of Health Care

**Figure 1.**
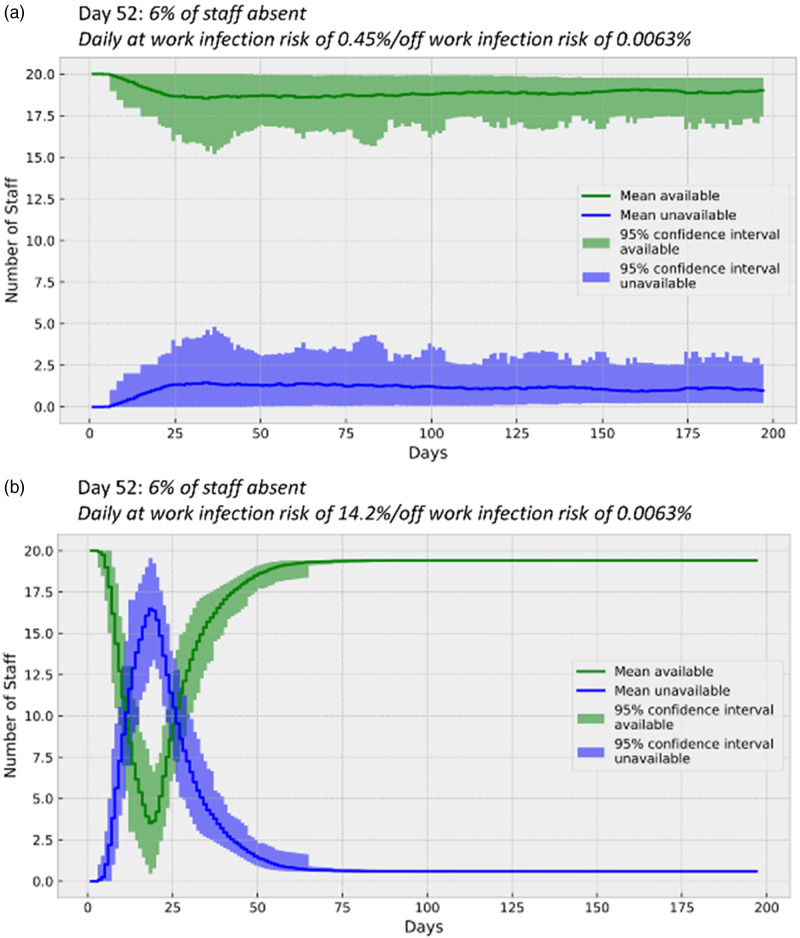
Different rates of personnel infection produce quoted UK health care absence figures. Simple models can be constructed to produce quoted health care staff infection statistics using constant daily infection rates (a and b). The model uses a Monte Carlo simulation to derive mean and confidence interval statistics.

Health care services are facing two simultaneous challenges: an unprecedented number of admissions and reductions in staff due to infection or self-isolation. An initial report from Wuhan, China, noted that 2% of those infected were health care workers.^1^ Early in 2020, media outlets were reporting large numbers of health care staff being infected in Europe; for example, 10 000 health staff were reported to be infected in Spain.^2^ In the UK, the president of the Royal College of Physicians was quoted as saying, “about one in four” of its workforce was unavailable,^3^ while the Secretary of State for Health and Social Care in the UK gave a figure of 5.7%^4^ for all National Health Service staff. As an example, these statistics can be reached, and very probably have been reached, in many ways (Figure 1). Since then, reports using data from many thousands of health care workers have noted a significantly increased risk of infection for this group.^5^

Traditional working patterns in hospitals are based on the historical workload of departments, and those tasked with their redesign are not used to the rates of staff absence being seen. We feel that this challenge would be better met if such information were made widely available. Reporting such figures, alongside the nature of the work environment (eg, intensive care), work pattern (ie, the rota), and personal protective equipment used would help health care leaders grapple with how to deploy their depleted ranks. There will be many innovative ways of working being employed, and these can benefit frontline staff both now and in the future.

We have created a simple online web application (https://covid19.shef.ac.uk) that can simulate staffing levels across different rota systems and infection rates. Simple and pseudocode descriptions are provided, and the python script is available in a GitHub repository (https://github.com/gyengen/Covid19-Rota-test). The app is aimed at those re-designing their services, providing a means to “stress test” a planned staffing strategy. Rather than offer fixed parameters, the user can vary infection rates and so simulate multiple scenarios. An option also exists to share the rota designs tested via the web application, although this is purely voluntary and does not preclude one from using the application. We hope to stimulate debate on this area and challenge the scientific community to create better ways of collecting/disseminating this information than our humble effort.
